# Pathogenesis of Thalassemia Major-associated Osteoporosis: A Review with Insights from Clinical Experience

**DOI:** 10.4274/jcrpe.galenos.2018.2018.0074

**Published:** 2019-05-28

**Authors:** Agostino Gaudio, Nancy Morabito, Antonino Catalano, Rosario Rapisarda, Anastasia Xourafa, Antonino Lasco

**Affiliations:** 1 University of Catania, Department of Clinical and Experimental Medicine, Catania, Italy; 2 University of Messina, Department of Clinical and Experimental Medicine, Messina, Italy

**Keywords:** Osteoporosis, thalassemia major, hypogonadism, marrow expansion, bone turnover

## Abstract

Due to increasing life expectancy in thalassemia major (TM), osteoporosis is emerging as a significant problem. Its aetiology is multifactorial, culminating in increased bone resorption and impaired remodelling. Hypogonadism and marrow expansion seem to play an important role, but iron overload, deferoxamine toxicity, a defective growth hormone-insulin-like growth factor-1 axis and multiple endocrinopathies may represent additional causes of bone damage. Many of these patients, though under appropriate treatment programs, do not achieve normal peak bone mass. The receptor activator of nuclear factor kappa-ß (RANK)/RANK ligand/osteoprotegerin and the Wnt/β-catenin systems work as major mediators of imbalanced bone turnover and bone loss. Additional genetic factors, such as collagen type 1 alpha 1 and vitamin D receptor gene polymorphisms, may exert some influence on the enhanced fracture risk observed in TM. To date, in spite of adequate hormone replacement, chelating therapy and acceptable haemoglobin levels, subjects with TM display impaired bone density and imbalanced bone turnover, thus the puzzle of the pathogenesis of TM-induced osteoporosis remains far from being solved.

## Introduction

Thalassemia major (TM) is a hereditary disease caused by defective globin synthesis, resulting in abnormal as well as a decreased quantity of globin chains, ineffective erythropoiesis, haemolysis and increased red blood cell turnover. Cooley et al (1) described the first patients with anaemia, splenomegaly and cranial and facial bone enlargement. These bone changes were due to the marked expansion of the bone marrow, secondary to anaemia and ineffective erythropoiesis (2,3). Although optimised blood transfusions and iron chelation programs have greatly increased the life expectancy of TM patients and prevented these severe bone alterations, osteoporosis and osteopenia remain serious complications, even in well-transfused and well-iron chelated patients (4).

The pathogenesis of bone changes in TM is not fully clarified. Several studies have shown that multiple factors may act in concert to produce bone disease in TM including bone marrow expansion (5), hypogonadism (6,7,8), defective growth hormone-insulin-like growth factor-1 (GH-IGF-1) axis (9,10,11,12), altered pattern of cytokines (13), iron deposit in bone (5,14,15), deferoxamine bone toxicity (16,17) and vitamin D deficiency (18). Some of these pathogenic factors, directly and/or indirectly, affect osteoblastic population, leading to depressed bone formation, while others often increase osteoclastic bone resorption.

In this review, in the light of our experience, we analysed the alterations of bone metabolism and the acquired and genetic factors that could be responsible for the development of osteopenia/osteoporosis in TM patients.

## Bone Metabolism in TM Patients

Osteoporosis is a skeletal disorder characterized by compromised bone strength, predisposing to an increased risk of fracture (19). According to the World Health Organization, diagnosis of osteoporosis is based on the T-score for bone mineral density (BMD), assessed at the lumbar spine or the femoral neck. Osteoporosis is defined by a BMD that is 2.5 standard deviations (SD) or more below the mean value for a young adult female (T-score less than or equal to -2.5 SD) (20). The Z-score describes the number of SDs by which the BMD in an individual differs from the mean value expected for a given age and sex. The diagnosis of osteoporosis in children and adolescents should not be made on the basis of densitometric criteria alone. In the absence of vertebral compression (crush) fractures, the diagnosis of osteoporosis is indicated by the presence of both a clinically significant fracture history and BMD Z-score less than or equal to -2.0 SD (21). In some studies (22,23), in order to reduce the influence of bone size on BMD measurements in the growing skeleton, the apparent volumetric density of the lumbar spine has been calculated using a specific formula (24).

In TM patients, it is very common to find low BMD values (osteopenia or osteoporosis) and in some studies up to 90%, even in optimally transfused and chelated patients, as is shown in Table 1 (8,25,26,27,28,29,30).

Prevalence of fractures in TM patients is depicted in Table 2 and ranges from 16% to 49%, depending on study population and method of data collection (3,31,32,33,34,35). Extremity fractures are the most common (26), in particular at the upper extremity (31). Vertebral fractures are usually underestimated, and their prevalence varies from 2.6% to 13% (26,36).

TM patients, in spite of following a regular transfusional regimen, and receiving adequate sex hormone replacement and chelating therapy, show imbalanced bone turnover with an increased resorptive phase that is not followed by an appropriate neoformation rate, resulting in a decreased BMD, particularly at the vertebral level, where trabecular bone is mostly represented (22,26,37,38,39). In previous studies (12,22), we described a decreased neoformation phase in accordance with Mahachoklertwattana et al (23) and histomorphometric studies performed by De Vernejoul et al (40).

The depression of bone formation, even if slight, is surprising because an increase in resorption is generally followed by a corresponding increase in bone formation due to coupling of bone turnover. Numerous acquired factors could lead to the inhibition of osteoblastic activity, such as a defective GH-IGF-1 axis, iron deposits in bone, or deferoxamine toxicity (12,22). Many studies (12,22,37,38,41,42) have shown increased osteoclast activation in these patients, measuring markers of bone resorption such as urinary levels of N-telopeptide of collagen type 1, serum levels of tartrate resistant acid phosphatase isoform-5b, and urinary pyridinium cross-links. The mechanism responsible for this osteoclast activation in well-treated thalassemic patients could be related to the altered cytokines network, which is often observed in these patients.

## Cytokines Network

The receptor activator of nuclear factor kappa-ß (RANK)/RANK ligand (RANKL)/osteoprotegerin (OPG) system regulates the activation and proliferation of osteoclast precursors (43). In our previous study (42), and in accordance with others (41), we found that the ratio of RANKL/OPG is increased in patients with TM and osteoporosis, showing that the RANKL/OPG system acts as an important paracrine mediator of bone metabolism also in TM patients. Cytokines other than RANKL and OPG, such as interleukin (IL)-1α, IL-6 and tumor necrosis factor-α, that are largely recognised as important effectors in the pathogenesis of several forms of osteoporosis (44,45,46,47), could have a role in TM-related osteoporosis. Our data (13) clearly showed an increase of circulating pro-osteoclastogenic cytokines associated with several markers of bone turnover and densitometric data, also pointing to their potential role in accelerating bone loss in TM-related osteoporosis. In particular, we observed significantly higher serum concentrations of IL-1α and IL-6 in TM patients and a significant correlation of these cytokines with urinary pyridinium cross-links (13).

Recently, the Wnt/β-catenin canonical pathway has been suggested to be involved in bone remodelling by promoting proliferation and differentiation of osteoblast precursor cells, reducing apoptosis of mature osteoblasts, and promoting the ability of differentiated osteoblasts to inhibit osteoclast differentiation. This pathway has been proposed to participate in the pathogenesis of osteoporosis in TM, and negative modulators of this signalling system, such as Dickkopf-1 and sclerostin, have also been associated with BMD in TM patients (48,49).

## Bone Marrow Expansion

Bone marrow expansion (2,3,4,50) is considered by various authors as a major determinant of bone destruction in TM patients. In spite of regular blood transfusions, the ineffective erythropoiesis in TM is not fully suppressed. Expansion of the bone marrow may contribute to the decreased BMD - even if data are contradictory (51) - because transferrin receptor studies have demonstrated increased bone marrow activity, even in patients with low reticulocyte count or marrow hypoplasia (52).

An intimate relationship between bone marrow and the process of remodelling exists, however. This interaction between bone marrow and bone tissue could explain the fact that bone loss in TM largely involves trabecular bone. In fact, the lumbar spine, which consists mostly of trabecular bone and with wide bone marrow spaces, is the most affected site in these patients (23).

It has been speculated that the increased generation of cells of the erythropoietic lineage may adversely affect the proliferation and maturation of cells of the osteogenic lineage. Osteoclasts originate from a hemopoietic granulocyte-macrophage lineage. The cytokines that are involved in haematopoiesis are also involved in the development of osteoclasts (53). Therefore, it is possible that the mechanism that stimulates haematopoiesis in TM may also stimulate osteoclastic formation and/or activity, which, in turn, increases bone resorption and reduces bone mass.

## Iron Overload in Endocrine Glands

A regular transfusional regimen is a cornerstone of TM treatment, but theis results in significant iron overload. Excessive iron is deposited in almost all tissues but primarily in the liver, the heart and the endocrine glands. Early introduction of a chelating agent to prevent iron overload in vulnerable organs leads to improved life expectancy (54).

TM patients often present with multiple endocrine dysfunctions including growth failure, hypogonadism, diabetes, hypothyroidism, hypoparathyroidism and, less frequently, hypoadrenalism (5,55,56,57). Several authors demonstrated that these abnormalities were closely related to iron overload, as shown by histological findings in different endocrine glands (58). Shamshirsaz et al (5), analysing 220 TM patients, found significant differences in mean serum ferritin levels between TM patients affected by primary amenorrhea and hypogonadism and TM patients without endocrinopathies. Moreover, the authors observed that impaired puberty was the most common endocrine abnormality (over 70% of the participants). The prevalence of other endocrinopathies was much lower with 17.5% hypogonadism, 8.7% diabetes mellitus, 7.7% primary hypothyroidism and 7.6% hypoparathyroidism. De Sanctis et al (55), analysed 1861 patients and reported slightly different data. In particular, failure of puberty was the major clinical endocrine defect and was present in 51% of boys and 47% of girls, all over the age of 15 years. Secondary amenorrhoea was recorded in 23% of patients, primary hypothyroidism in 6.2%, insulin dependent diabetes mellitus in 4.9% and hypoparathyroidism in 3.6% of the patients.

## Hypogonadism

Although data on prevalence are discordant, as reported above, TM patients often show gonadal impairment (6). Haemosiderosis of the pituitary gonadotrophic cells and iron deposition in the testes and ovaries are involved in the pathogenesis of hypogonadism in TM (59,60). In addition hypogonadism is a well-recognised cause of osteoporosis and osteopenia, not only in TM, but also in the general population (61,62,63).

In our previous study (22), in accordance with Anapliotou et al (6) and Jensen et al (8), we showed that hypogonadism produces more severe bone loss in TM. Our group had already shown that TM patients complained of various degrees of osteopenia due to their hormonal status. In fact, we observed that in TM patients without evidence of hypogonadism because of hormone replacement therapy, bone status was less compromised and osteoporosis was observed only at the lumbar site, where the influence of bone marrow expansion is prominent, as described above. However, in hypogonadic patients, osteoporosis may be more severe and may also affect the femoral site. Furthermore, we found a significant positive correlation between BMD values and hormonal treatment duration.

## GH-IGF-1 Axis

Several studies showed that the GH-IGF-1 axis is altered in TM patients (11,12). These patients have significantly lower circulating levels of IGF-1 and the corresponding binding protein (IGFBP-3) than normal individuals (11,12). IGF-1 plays an important role in bone remodelling. Low serum IGF levels decrease osteoblast proliferation and bone matrix formation and reduce the activation of osteoclasts (64). A positive correlation between BMD at the lumbar spine and IGF-1 concentration has been reported (48,65). In our previous work (12), we found lower serum levels of IGF-l and IGFBP-3 in TM patients than in age-matched, healthy controls and a significant correlation between IGF-1, osteocalcin which is a marker of bone formation, and BMD values. Similarly, low concentrations of IGF-1 in TM adults and their correlation with BMD have been reported by Dresner Pollak et al (37).

The mechanisms responsible for the reduced action of the IGF-1/IGFBP-3 axis in TM are still being debated. Danesi et al (66) found an impairment of GH secretion in a considerable proportion of TM patients, compatible with hypothalamic and/or pituitary damage. It is unclear whether the IGF-1 level decreases before or after GH secretion dysfunction (67,68,69). Chrysis et al (70) suggested that impaired GH secretion, rather than GH insensitivity, is the cause of growth retardation in TM patients.

## Iron Deposition in Bone

Iron deposition in bone damages osteoid maturation and inhibits mineralisation, resulting in focal osteomalacia. This is due to the incorporation of iron into crystals of calcium hydroxyapatite, which consequently affects the growth of hydroxyapatite crystals and reduces basic multicellular unit tensile strength (71). Mahachoklertwattana et al (23) observed increased osteoid thickness, osteoid maturation time and mineralisation lag time in TM patients.

## Deferoxamine

Subcutaneously administered deferoxamine was for a long time the treatment of choice for iron overload in TM. Its chelating action is not solely specific for iron. Deferoxamine also inhibits DNA synthesis, collagen formation and osteoblast precursor differentiation and enhances osteoblast apoptosis (16,17). Data on bone safety of new oral chelating agents are still limited.

## Vitamin D

Vitamin D deficiency is involved in the pathogenesis of osteoporosis in TM patients due to its regulatory effects on bone cells and calcium homeostasis. Lower 25-hydroxyvitamin D levels, in comparison to healthy controls, are a common finding and are inversely correlated with ferritin levels and age. Lower sun exposure due to reduced physical activity and defective skin synthesis associated with jaundice are probably responsible for this deficiency (72).

## Genetic Factors

Genetic factors also have an important role in determining BMD in TM patients, although the genes responsible are poorly defined in this population. Some studies provide partially support for an association between BMD and specific *COL1A1* (73) and *TGF-*β*1* (74) gene polymorphism in TM. Vitamin D receptor (VDR) polymorphisms could also represent a risk factor for low BMD in adult TM patients (37,75). In our thalassemic population, we found that VDR (FokI, BsmI) and *COL1A1* (Sp1) gene polymorphisms had no influence on BMD, but BsmI was found to display beneficial effects on patient response to alendronate therapy (76). It has recently been reported that the f allele of the *Fok-I* gene polymorphism, when found in homozygosity, confers protection on the BMD values of young thalassemic patients (77).

## Conclusion

**Figure 1:**
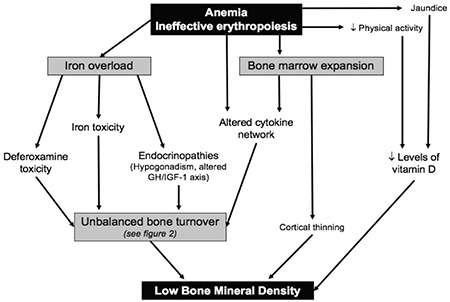
Pathogenesis of low bone mineral density in thalassemic patients GH-IGF-1: growth hormone-insulin-like growth factor-1

**Figure 2:**
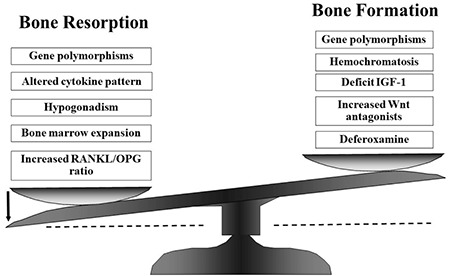
Possible causes for uncoupling bone turnover in thalassemic patients IGF-1: insulin-like growth factor-1, RANKL/OPG: receptor activator of nuclear factor kappa-ß/osteoprotegerin

Multiple acquired factors, together with genetic variants that predispose individuals to reduced BMD, contribute to bone fragility in TM. Bone marrow expansion, hypogonadism, a defective GH-IGF-1 axis and imbalanced cytokine profiles play major roles in the development of osteoporosis. Iron overload, deferoxamine toxicity and other endocrine dysfunctions could be additional factors. Figures 1 and 2 summarise potential factors contributing to the imbalanced bone turnover in TM patients. To date, in spite of adequate hormone replacement therapy, acceptable haemoglobin levels and chelating therapy, TM patients unexpectedly display impaired BMD and imbalanced bone turnover, indicating that the puzzle of the pathogenesis of TM-related osteoporosis is still far from being fully solved.

## Figures and Tables

**Table 1:**
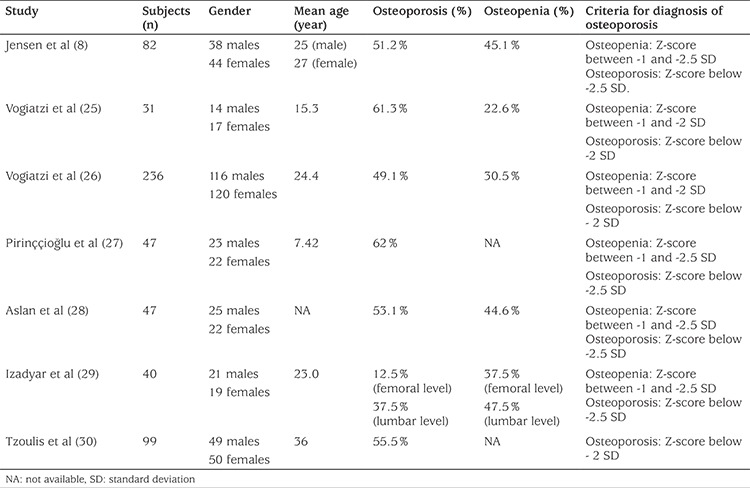
Osteoporosis/osteopenia prevalence in thalassemia major patients

**Table 2:**
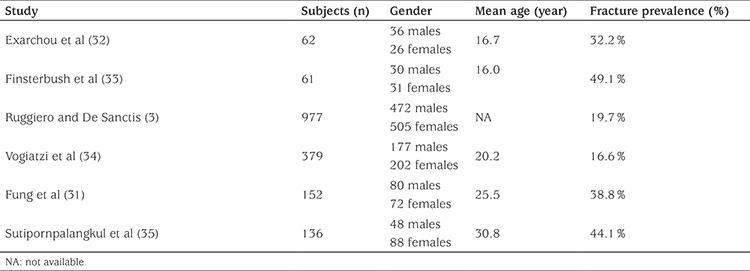
Fracture prevalence in thalassemia major patients
